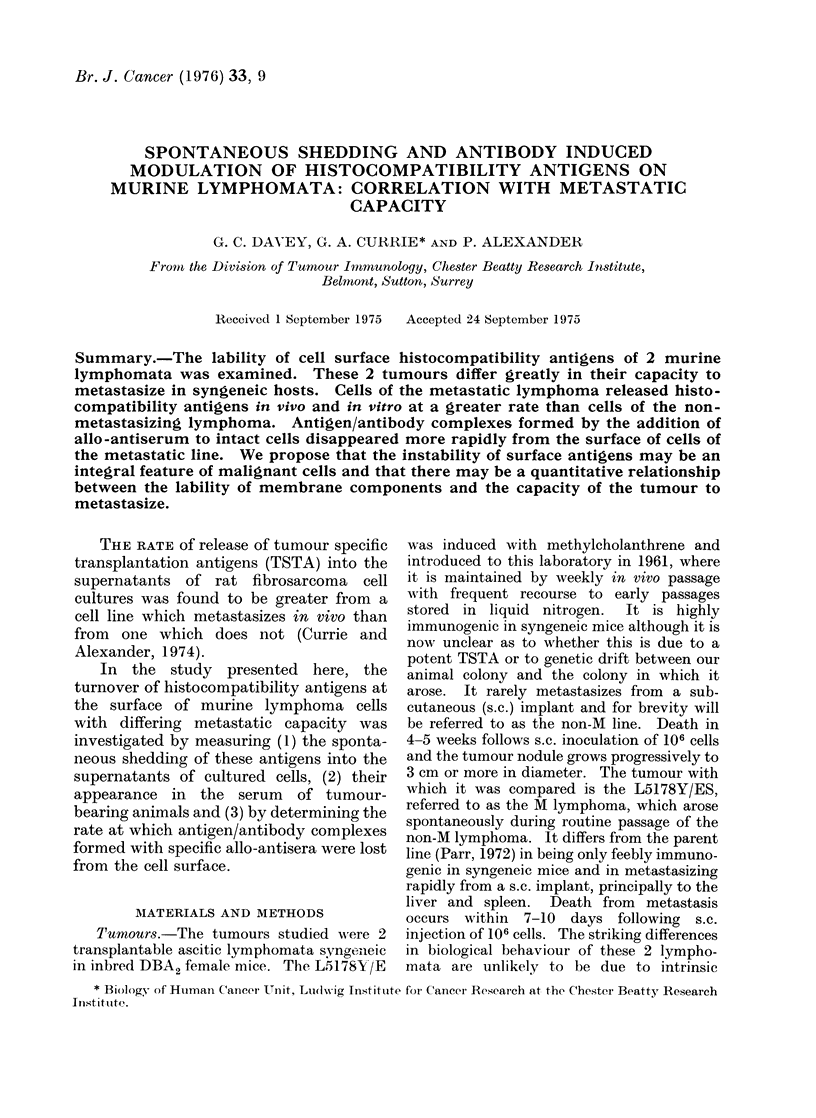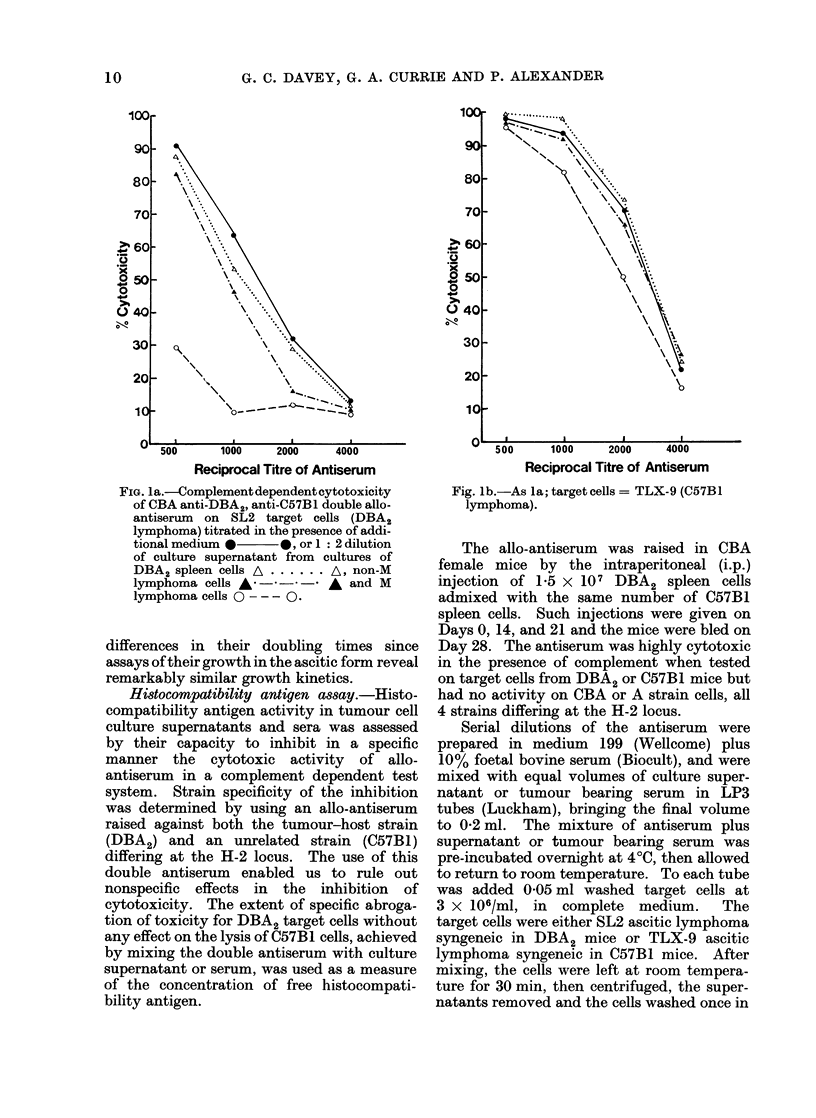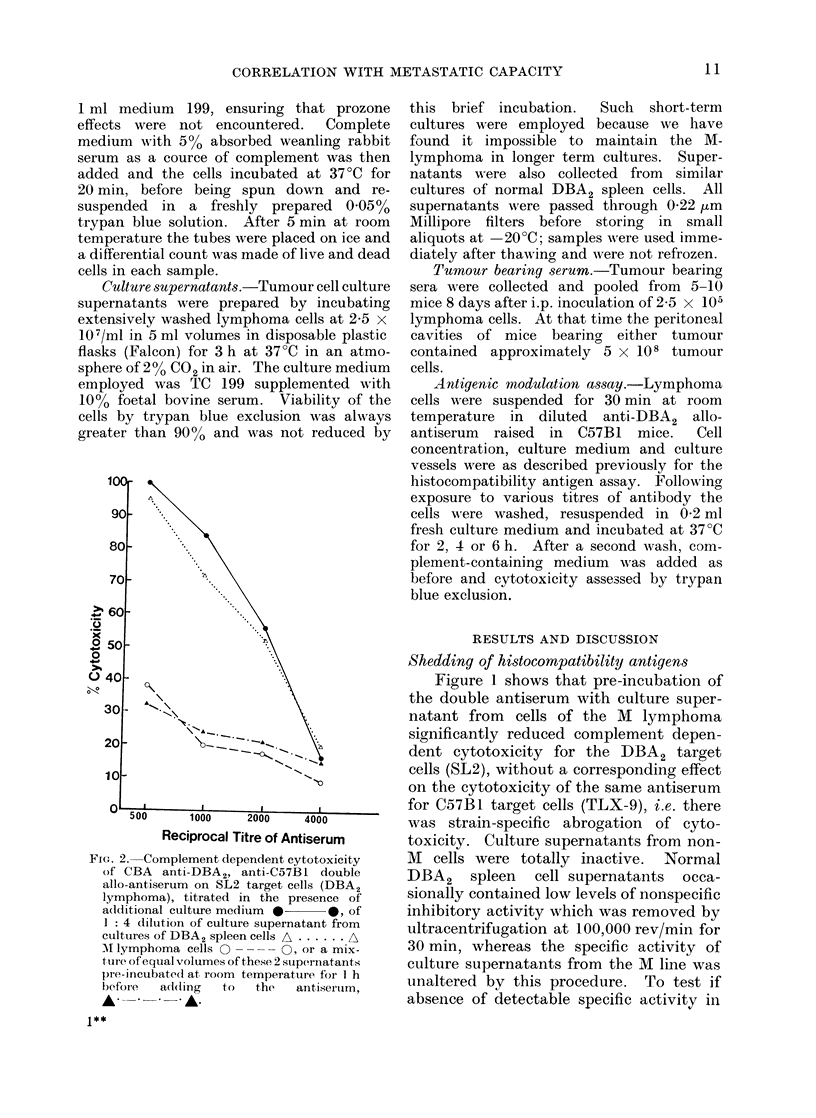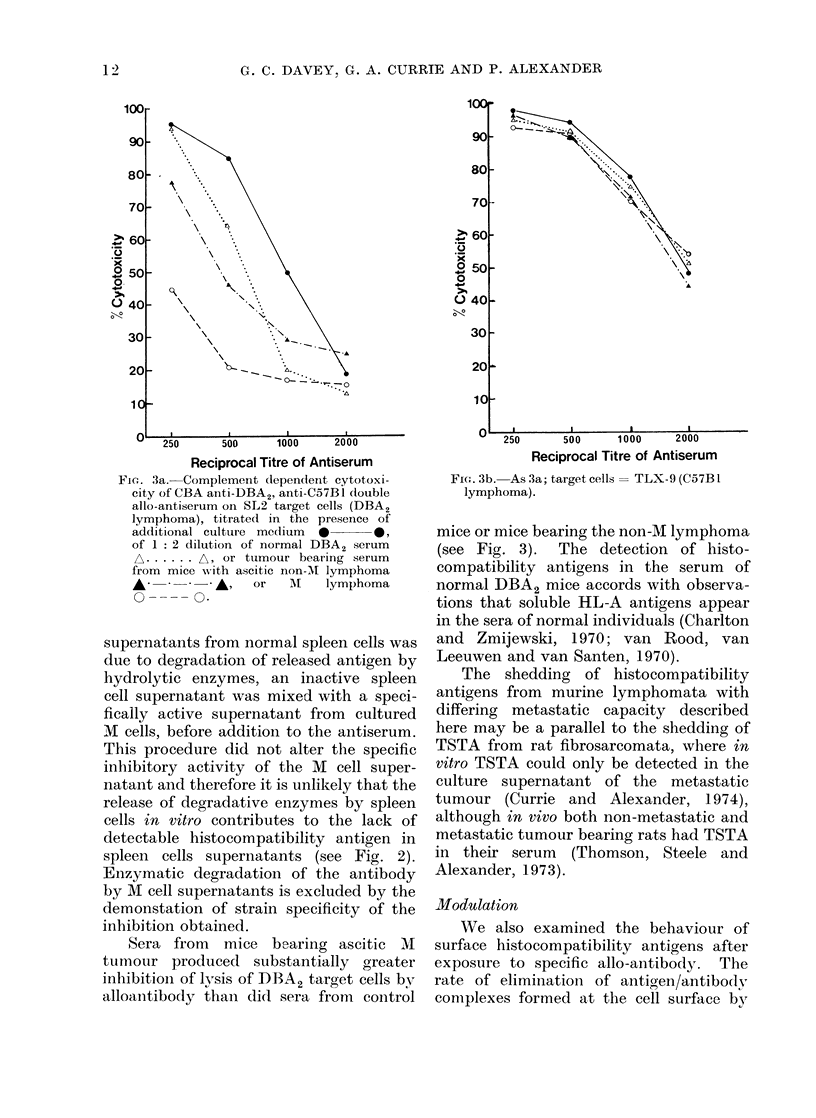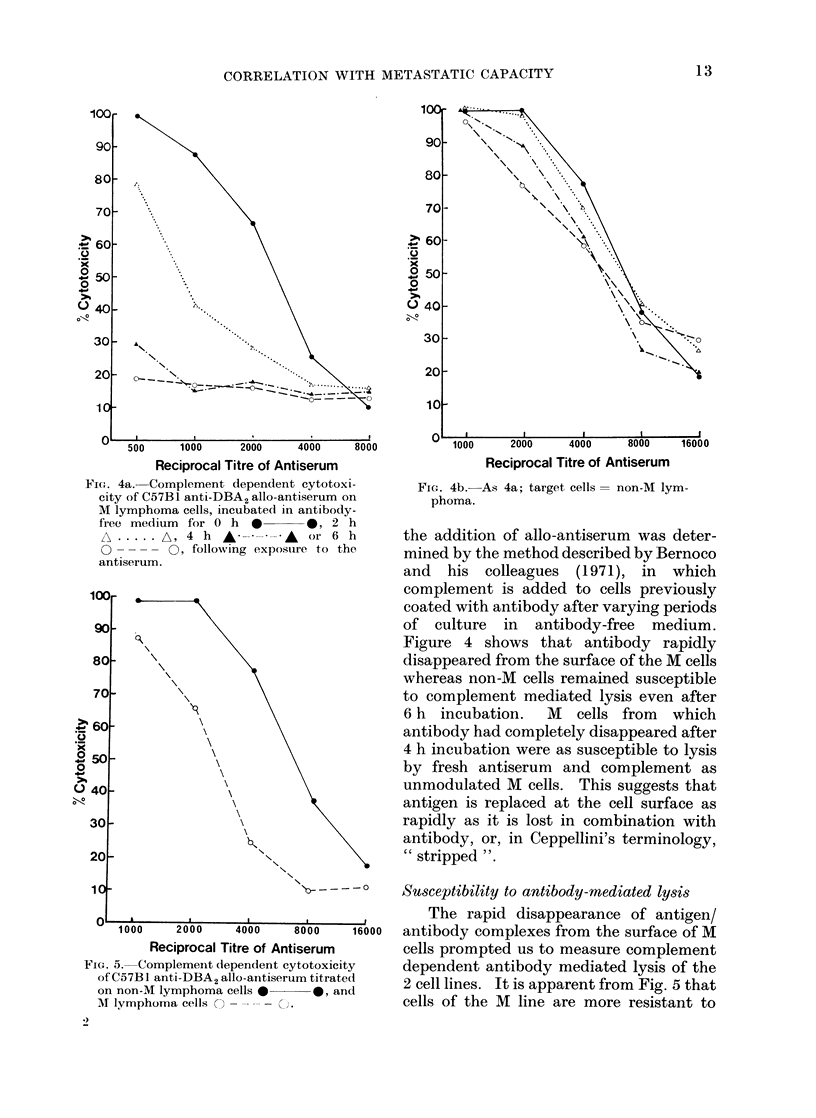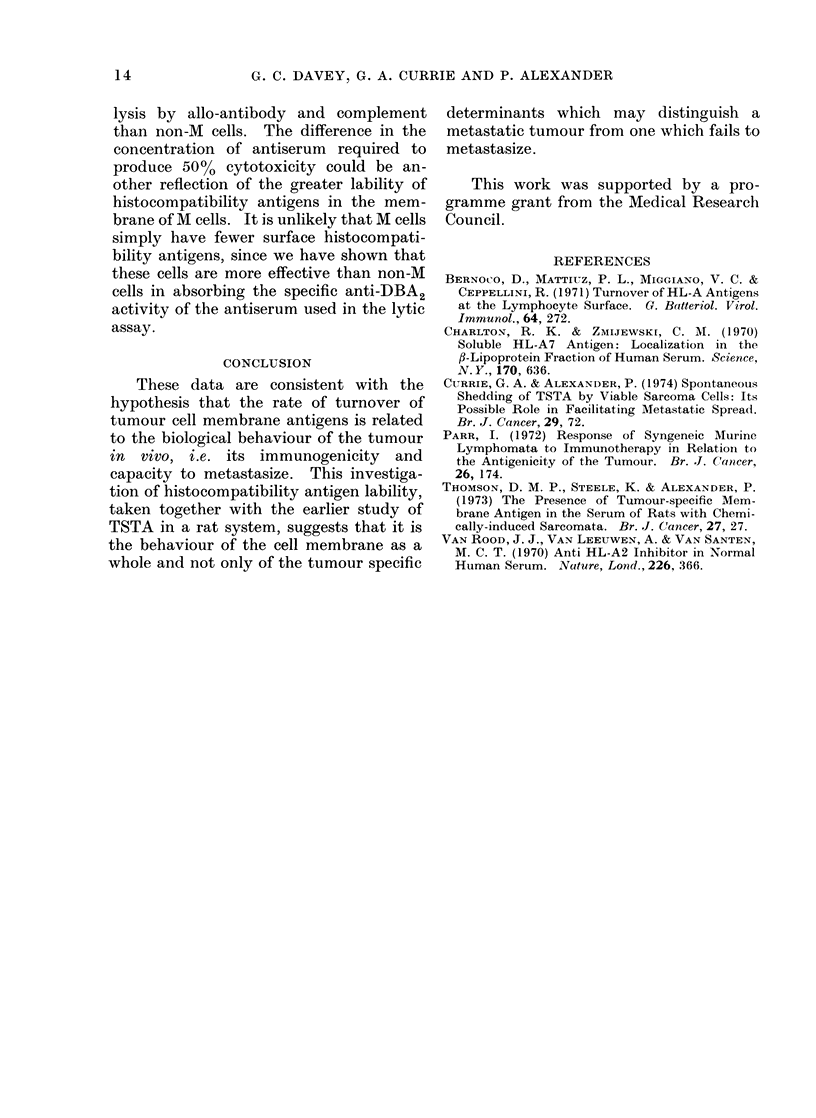# Spontaneous shedding and antibody induced modulation of histocompatibility antigens on murine lymphomata: Correlation with metastic capacity.

**DOI:** 10.1038/bjc.1976.2

**Published:** 1976-01

**Authors:** G. C. Davey, G. A. Currie, P. Alexander

## Abstract

The lability of cell surface histocompatibility antigens of 2 murine lymphomata was examined. These 2 tumours differ greatly in their capacity to metastasize in syngeneic hosts. Cells of the metastatic lymphoma released histocompatibility antigens in vivo and in vitro at a greater rate than cells of the non-metastasizing lymphoma. Antigen/antibody complexes formed by the addition of allo-antiserum to intact cells disappeared more rapidly from the surface of cells of the metastatic line. We propose that the instability of surface antigens may be an integral feature of malignant cells and that there may be a quantitative relationship between the lability of membrane components and the capacity of the tumour to metastasize.


*Br. J. Cancer* (1976) **33**, 9

### SPONTANEOUS SHEDDING AND ANTIBODY INDUCED MODULATION OF HISTOCOMPATIBILITY ANTIGENS ON MURINE LYMPHOMATA: CORRELATION WITH METASTATIC CAPACITY

G. C. DAVEY, G. A. CURRIE* AND P. ALEXANDER

*From the Division of Tumour Immunology, Chester Beatty Research Institute, Belmont, Sutton, Surrey*

Received 1 September 1975 Accepted 24 September 1975

**Summary.—The lability of cell surface histocompatibility antigens of 2 murine lymphomata was examined. These 2 tumours differ greatly in their capacity to metastasize in syngeneic hosts. Cells of the metastatic lymphoma released histocompatibility antigens *in vivo* and *in vitro* at a greater rate than cells of the non-metastasizing lymphoma. Antigen/antibody complexes formed by the addition of allo-antiserum to intact cells disappeared more rapidly from the surface of cells of the metastatic line. We propose that the instability of surface antigens may be an integral feature of malignant cells and that there may be a quantitative relationship between the lability of membrane components and the capacity of the tumour to metastasize.**

THE RATE of release of tumour specific transplantation antigens (TSTA) into the supernatants of rat fibrosarcoma cell cultures was found to be greater from a cell line which metastasizes *in vivo* than from one which does not (Currie and Alexander, 1974).

In the study presented here, the turnover of histocompatibility antigens at the surface of murine lymphoma cells with differing metastatic capacity was investigated by measuring (1) the spontaneous shedding of these antigens into the supernatants of cultured cells, (2) their appearance in the serum of tumour-bearing animals and (3) by determining the rate at which antigen/antibody complexes formed with specific allo-antisera were lost from the cell surface.

### MATERIALS AND METHODS

*Tumours.*—The tumours studied were 2 transplantable ascitic lymphomata syngeneic in inbred $DBA_2$ female mice. The L5178Y/E was induced with methylcholanthrene and introduced to this laboratory in 1961, where it is maintained by weekly *in vivo* passage with frequent recourse to early passages stored in liquid nitrogen. It is highly immunogenic in syngeneic mice although it is now unclear as to whether this is due to a potent TSTA or to genetic drift between our animal colony and the colony in which it arose. It rarely metastasizes from a subcutaneous (s.c.) implant and for brevity will be referred to as the non-M line. Death in 4–5 weeks follows s.c. inoculation of $10^6$ cells and the tumour nodule grows progressively to 3 cm or more in diameter. The tumour with which it was compared is the L5178Y/ES, referred to as the M lymphoma, which arose spontaneously during routine passage of the non-M lymphoma. It differs from the parent line (Parr, 1972) in being only feebly immunogenic in syngeneic mice and in metastasizing rapidly from a s.c. implant, principally to the liver and spleen. Death from metastasis occurs within 7–10 days following s.c. injection of $10^6$ cells. The striking differences in biological behaviour of these 2 lymphomata are unlikely to be due to intrinsic

* Biology of Human Cancer Unit, Ludwig Institute for Cancer Research at the Chester Beatty Research Institute.

FIG. 1a.—Complement dependent cytotoxicity of CBA anti-$DBA_2$, anti-C57Bl double allo-antiserum on SL2 target cells ($DBA_2$ lymphoma) titrated in the presence of additional medium ●———●, or 1 : 2 dilution of culture supernatant from cultures of $DBA_2$ spleen cells △ . . . . . . △, non-M lymphoma cells ▲·—·—·—· ▲ and M lymphoma cells ○ – – – ○.

Fig. 1b.—As 1a; target cells = TLX-9 (C57Bl lymphoma).

differences in their doubling times since assays of their growth in the ascitic form reveal remarkably similar growth kinetics.

*Histocompatibility antigen assay.*—Histocompatibility antigen activity in tumour cell culture supernatants and sera was assessed by their capacity to inhibit in a specific manner the cytotoxic activity of allo-antiserum in a complement dependent test system. Strain specificity of the inhibition was determined by using an allo-antiserum raised against both the tumour–host strain ($DBA_2$) and an unrelated strain (C57Bl) differing at the H-2 locus. The use of this double antiserum enabled us to rule out nonspecific effects in the inhibition of cytotoxicity. The extent of specific abrogation of toxicity for $DBA_2$ target cells without any effect on the lysis of C57Bl cells, achieved by mixing the double antiserum with culture supernatant or serum, was used as a measure of the concentration of free histocompatibility antigen.

The allo-antiserum was raised in CBA female mice by the intraperitoneal (i.p.) injection of $1{\cdot}5 \times 10^7$ $DBA_2$ spleen cells admixed with the same number of C57Bl spleen cells. Such injections were given on Days 0, 14, and 21 and the mice were bled on Day 28. The antiserum was highly cytotoxic in the presence of complement when tested on target cells from $DBA_2$ or C57Bl mice but had no activity on CBA or A strain cells, all 4 strains differing at the H-2 locus.

Serial dilutions of the antiserum were prepared in medium 199 (Wellcome) plus 10% foetal bovine serum (Biocult), and were mixed with equal volumes of culture supernatant or tumour bearing serum in LP3 tubes (Luckham), bringing the final volume to 0·2 ml. The mixture of antiserum plus supernatant or tumour bearing serum was pre-incubated overnight at 4°C, then allowed to return to room temperature. To each tube was added 0·05 ml washed target cells at $3 \times 10^6$/ml, in complete medium. The target cells were either SL2 ascitic lymphoma syngeneic in $DBA_2$ mice or TLX-9 ascitic lymphoma syngeneic in C57Bl mice. After mixing, the cells were left at room temperature for 30 min, then centrifuged, the supernatants removed and the cells washed once in

1 ml medium 199, ensuring that prozone effects were not encountered. Complete medium with 5% absorbed weanling rabbit serum as a cource of complement was then added and the cells incubated at 37°C for 20 min, before being spun down and resuspended in a freshly prepared 0·05% trypan blue solution. After 5 min at room temperature the tubes were placed on ice and a differential count was made of live and dead cells in each sample.

*Culture supernatants.*—Tumour cell culture supernatants were prepared by incubating extensively washed lymphoma cells at $2{\cdot}5 \times 10^7$/ml in 5 ml volumes in disposable plastic flasks (Falcon) for 3 h at 37°C in an atmosphere of 2% $CO_2$ in air. The culture medium employed was TC 199 supplemented with 10% foetal bovine serum. Viability of the cells by trypan blue exclusion was always greater than 90% and was not reduced by this brief incubation. Such short-term cultures were employed because we have found it impossible to maintain the M-lymphoma in longer term cultures. Supernatants were also collected from similar cultures of normal $DBA_2$ spleen cells. All supernatants were passed through 0·22 μm Millipore filters before storing in small aliquots at −20°C; samples were used immediately after thawing and were not refrozen.

FIG. 2.—Complement dependent cytotoxicity of CBA anti-$DBA_2$, anti-C57B1 double allo-antiserum on SL2 target cells ($DBA_2$ lymphoma), titrated in the presence of additional culture medium ●———●, of 1 : 4 dilution of culture supernatant from cultures of $DBA_2$ spleen cells △ . . . . . . △ M lymphoma cells ○ – – – – ○, or a mixture of equal volumes of these 2 supernatants pre-incubated at room temperature for 1 h before adding to the antiserum, ▲·—·—·—·▲.

*Tumour bearing serum.*—Tumour bearing sera were collected and pooled from 5–10 mice 8 days after i.p. inoculation of $2{\cdot}5 \times 10^5$ lymphoma cells. At that time the peritoneal cavities of mice bearing either tumour contained approximately $5 \times 10^8$ tumour cells.

*Antigenic modulation assay.*—Lymphoma cells were suspended for 30 min at room temperature in diluted anti-$DBA_2$ allo-antiserum raised in C57B1 mice. Cell concentration, culture medium and culture vessels were as described previously for the histocompatibility antigen assay. Following exposure to various titres of antibody the cells were washed, resuspended in 0·2 ml fresh culture medium and incubated at 37°C for 2, 4 or 6 h. After a second wash, complement-containing medium was added as before and cytotoxicity assessed by trypan blue exclusion.

### RESULTS AND DISCUSSION

### *Shedding of histocompatibility antigens*

Figure 1 shows that pre-incubation of the double antiserum with culture supernatant from cells of the M lymphoma significantly reduced complement dependent cytotoxicity for the $DBA_2$ target cells (SL2), without a corresponding effect on the cytotoxicity of the same antiserum for C57B1 target cells (TLX-9), *i.e.* there was strain-specific abrogation of cytotoxicity. Culture supernatants from non-M cells were totally inactive. Normal $DBA_2$ spleen cell supernatants occasionally contained low levels of nonspecific inhibitory activity which was removed by ultracentrifugation at 100,000 rev/min for 30 min, whereas the specific activity of culture supernatants from the M line was unaltered by this procedure. To test if absence of detectable specific activity in

FIG. 3a.—Complement dependent cytotoxicity of CBA anti-$DBA_2$, anti-C57Bl double allo-antiserum on SL2 target cells ($DBA_2$ lymphoma), titrated in the presence of additional culture medium ●———●, of 1 : 2 dilution of normal $DBA_2$ serum △······△, or tumour bearing serum from mice with ascitic non-M lymphoma ▲·—·—·—·▲, or M lymphoma ○----○.

FIG. 3b.—As 3a; target cells = TLX-9 (C57Bl lymphoma).

supernatants from normal spleen cells was due to degradation of released antigen by hydrolytic enzymes, an inactive spleen cell supernatant was mixed with a specifically active supernatant from cultured M cells, before addition to the antiserum. This procedure did not alter the specific inhibitory activity of the M cell supernatant and therefore it is unlikely that the release of degradative enzymes by spleen cells *in vitro* contributes to the lack of detectable histocompatibility antigen in spleen cells supernatants (see Fig. 2). Enzymatic degradation of the antibody by M cell supernatants is excluded by the demonstation of strain specificity of the inhibition obtained.

Sera from mice bearing ascitic M tumour produced substantially greater inhibition of lysis of $DBA_2$ target cells by alloantibody than did sera from control mice or mice bearing the non-M lymphoma (see Fig. 3). The detection of histocompatibility antigens in the serum of normal $DBA_2$ mice accords with observations that soluble HL-A antigens appear in the sera of normal individuals (Charlton and Zmijewski, 1970; van Rood, van Leeuwen and van Santen, 1970).

The shedding of histocompatibility antigens from murine lymphomata with differing metastatic capacity described here may be a parallel to the shedding of TSTA from rat fibrosarcomata, where *in vitro* TSTA could only be detected in the culture supernatant of the metastatic tumour (Currie and Alexander, 1974), although *in vivo* both non-metastatic and metastatic tumour bearing rats had TSTA in their serum (Thomson, Steele and Alexander, 1973).

*Modulation*

We also examined the behaviour of surface histocompatibility antigens after exposure to specific allo-antibody. The rate of elimination of antigen/antibody complexes formed at the cell surface by

FIG. 4a.—Complement dependent cytotoxicity of C57Bl anti-$DBA_2$ allo-antiserum on M lymphoma cells, incubated in antibody-free medium for 0 h ●———●, 2 h △ . . . . . △, 4 h ▲·—·—·▲ or 6 h ○ – – – – ○, following exposure to the antiserum.

FIG. 4b.—As 4a; target cells = non-M lymphoma.

FIG. 5.—Complement dependent cytotoxicity of C57Bl anti-$DBA_2$ allo-antiserum titrated on non-M lymphoma cells ●———●, and M lymphoma cells ○ – – – – ○.

the addition of allo-antiserum was determined by the method described by Bernoco and his colleagues (1971), in which complement is added to cells previously coated with antibody after varying periods of culture in antibody-free medium. Figure 4 shows that antibody rapidly disappeared from the surface of the M cells whereas non-M cells remained susceptible to complement mediated lysis even after 6 h incubation. M cells from which antibody had completely disappeared after 4 h incubation were as susceptible to lysis by fresh antiserum and complement as unmodulated M cells. This suggests that antigen is replaced at the cell surface as rapidly as it is lost in combination with antibody, or, in Ceppellini's terminology, " stripped ".

*Susceptibility to antibody-mediated lysis*

The rapid disappearance of antigen/antibody complexes from the surface of M cells prompted us to measure complement dependent antibody mediated lysis of the 2 cell lines. It is apparent from Fig. 5 that cells of the M line are more resistant to lysis by allo-antibody and complement than non-M cells. The difference in the concentration of antiserum required to produce 50% cytotoxicity could be another reflection of the greater lability of histocompatibility antigens in the membrane of M cells. It is unlikely that M cells simply have fewer surface histocompatibility antigens, since we have shown that these cells are more effective than non-M cells in absorbing the specific anti-$DBA_2$ activity of the antiserum used in the lytic assay.

### CONCLUSION

These data are consistent with the hypothesis that the rate of turnover of tumour cell membrane antigens is related to the biological behaviour of the tumour *in vivo*, *i.e.* its immunogenicity and capacity to metastasize. This investigation of histocompatibility antigen lability, taken together with the earlier study of TSTA in a rat system, suggests that it is the behaviour of the cell membrane as a whole and not only of the tumour specific determinants which may distinguish a metastatic tumour from one which fails to metastasize.

This work was supported by a programme grant from the Medical Research Council.

### REFERENCES

BERNOCO, D., MATTIUZ, P. L., MIGGIANO, V. C. & CEPPELLINI, R. (1971) Turnover of HL-A Antigens at the Lymphocyte Surface. *G. Batteriol. Virol. Immunol.*, **64**, 272.

CHARLTON, R. K. & ZMIJEWSKI, C. M. (1970) Soluble HL-A7 Antigen: Localization in the $\beta$-Lipoprotein Fraction of Human Serum. *Science, N.Y.*, **170**, 636.

CURRIE, G. A. & ALEXANDER, P. (1974) Spontaneous Shedding of TSTA by Viable Sarcoma Cells: Its Possible Role in Facilitating Metastatic Spread. *Br. J. Cancer*, **29**, 72.

PARR, I. (1972) Response of Syngeneic Murine Lymphomata to Immunotherapy in Relation to the Antigenicity of the Tumour. *Br. J. Cancer*, **26**, 174.

THOMSON, D. M. P., STEELE, K. & ALEXANDER, P. (1973) The Presence of Tumour-specific Membrane Antigen in the Serum of Rats with Chemically-induced Sarcomata. *Br. J. Cancer*, **27**, 27.

VAN ROOD, J. J., VAN LEEUWEN, A. & VAN SANTEN, M. C. T. (1970) Anti HL-A2 Inhibitor in Normal Human Serum. *Nature, Lond.*, **226**, 366.